# Draft Genome Sequence of *Dietzia* sp. Strain SYD-A1, Isolated from Coal Seam Formation Water

**DOI:** 10.1128/MRA.01341-20

**Published:** 2021-03-11

**Authors:** Silas H. W. Vick, Paul Greenfield, Sasha G. Tetu, David J. Midgley, Ian T. Paulsen

**Affiliations:** a Department of Molecular Sciences, Macquarie University, North Ryde, Australia; b Commonwealth Scientific and Industrial Research Organisation (CSIRO), North Ryde, Australia; c Faculty of Chemistry, Biotechnology, and Food Science, Norwegian University of Life Sciences, Ås, Norway; Georgia Institute of Technology

## Abstract

Subsurface coal seams harbor an array of diverse microbial species subsisting as a community on the organic matter present in coal. Here, we present the annotated genome sequence of *Dietzia* sp. strain SYD-A1, a bacterium isolated from a terrestrial subsurface coal seam in New South Wales, Australia.

## Abstract (precis)

Subsurface coal seams harbor an array of diverse microbial species subsisting as a community on the organic matter present in coal. Here, we present the annotated genome sequence of *Dietzia* sp. strain SYD-A1, a bacterium isolated from a terrestrial subsurface coal seam in New South Wales (NSW), Australia.

## ANNOUNCEMENT

Microbial communities in coal seams are responsible for the transformation of fossilized coal organic matter to methane, which can then reenter the atmosphere or biosphere. This process is of interest to industry, as liberated methane can be used as a fuel source for energy generation, as well as to our understanding of global carbon cycling. As such, microbes in subsurface coal seams, as well as the genomes they harbor, are critical to our understanding of how this biogeochemical transformation is carried out.

Subsurface coal seams in eastern Australia are host to an array of microbial taxa. A sample of anoxic formation water from a Sydney Basin coal seam gas well (Sydney Basin well 2 [1]) was obtained from a depth of ∼650 m and subjected to enrichment culturing and isolation. One of these isolation strategies sought to obtain facultatively aerobic taxa by plating 50 μl of formation water onto tryptone soy agar (TSA) plates and incubating this at reservoir temperature (37°C) for 72 h. Colonies were picked and streaked onto TSA plates until an axenic culture of a Gram-positive, nonmotile, short (0.8 to 1.0 by 1.0 to 2.2 μm) rod-shaped bacterium was obtained.

**FIG 1:**
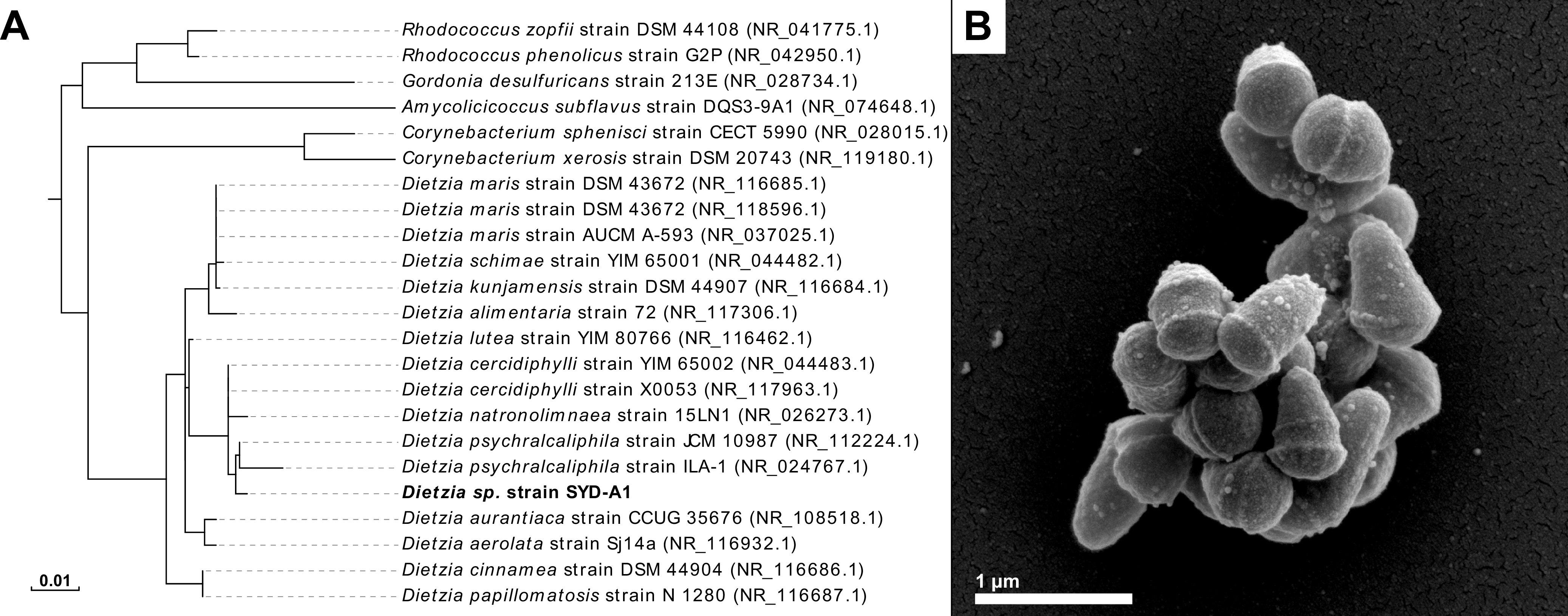
(A) Phylogenetic analysis of *Dietzia* species and outgroup organisms based on near-full-length 16S rRNA gene sequences obtained from the GenBank database. The 16S rRNA gene sequence for *Dietzia* sp. SYD-A1 was obtained from the whole-genome sequence of *Dietzia* sp. SYD-A1 presented here. Phylogenetic analysis was performed using the phylogeny.fr “one-click analysis” (http://www.phylogeny.fr/) (9). This includes alignment with MUSCLE v3.8.31 (10), refinement with Gblocks v0.91b (11), maximum likelihood phylogeny estimation with PhyML v3.1/3.0 aLRT (12, 13), and tree rendering with TreeDyn v198.3 (14). The scale bar for branch lengths indicates the number of substitutions per site. (B) Scanning electron microscopy image of *Dietzia* sp. SYD-A1; bar, 1 μm. Sample preparation and imaging were performed as previously described (15).

Genomic DNA was extracted from cells grown aerobically in tryptone soya broth (37°C) using the FastDNA spin kit (MP Biomedicals) according to the manufacturer’s instructions and subjected to library preparation using the Nextera XT kit and subsequent paired-end (250 bp) Illumina sequencing (The Ramaciotti Centre for Genomics, University of New South Wales, Australia). The resultant reads (7,457,466 paired-end reads) were corrected using Blue v1 (2) prior to assembly with Velvet v1.2.10 (3). The draft genome sequence was ∼3.6 Mbp long and comprised 595 contigs with a kmer coverage of ∼64× and a GC content of 68.9%. The mean, median, and *N*_50_ lengths for the assembly were 5,990, 4,038, and 9,280 bp, respectively. The genome was annotated using the IMG-ER pipeline v4.0 and contains 3,676 putative protein-coding genes (4). BLASTn v2.7.1 comparison of the annotated 16S rRNA gene against the NCBI reference sequence database identified the sequence as most closely related to that of members of the genus *Dietzia*. Further phylogenetic analysis is outlined in Fig. 1, and the isolate was termed *Dietzia* sp. strain SYD-A1. Comparison of the annotated 16S rRNA gene sequence to a previously published database of coal seam-derived operational taxonomic units (OTUs) matched *Dietzia* sp. SYD-A1 to the OTU CSMB200 (1).

In order to better understand the potential metabolism of the strain, the genome was submitted to the TransportDB v2.0 (http://membranetransport.org/transportDB2/) and dbCAN v7 (http://bcb.unl.edu/dbCAN2/) Web servers to interrogate its transporters and carbohydrate active enzymes (5, 6). The genome encodes a modest number of carbohydrate active enzymes that appear to be transported to the cell surface via signal peptides. These include representatives with probable β-glucosidase activities (glycoside hydrolase 1 [GH1] and GH3), arabino/xylanosidase activity (GH43), and cellulolytic activity (GH5), along with a number of gene families with activity toward peptidoglycan (GH23 and GH25) (7). These latter glycoside hydrolases may be used for internal cell wall reorganization or have roles in scavenging carbon from peptidoglycan from moribund cellular material. Alkane degradation has been shown to be a key metabolic pathway for *Dietzia* species from petroleum-contaminated environments and is mediated by the alkane hydroxylase gene *alkB* and two cytochrome P450 CYP153 family genes (8). The presence of *alkB* and the two CYP153 genes in the genome of *Dietzia* sp. SYD-A1 was confirmed with BLASTn v2.7.1 matching of gene sequences from *Dietzia* sp. strain H0B to the *Dietzia* sp. strain SYD-A1 genome (*alkB* [GenBank accession number FJ435355.1], CYP153_1 [FJ435360.1], and CYP153_2 [FJ435362.1]). The presence of these genes suggests that SYD-A1 is capable of utilizing coal-derived alkanes during periods of oxic exposure in the terrestrial subsurface. Culture-based growth experiments on a range of substrates would be useful for clarifying the roles of these catabolic genes *in situ*. Default parameters were used for all software analyses unless otherwise stated.

### Data availability.

This whole-genome sequence has been deposited in GenBank and is available under the accession number JADDKI000000000. The raw sequencing reads are available from the Sequence Read Archive under accession number SRR12786059.
